# Traité de Vaccine, sur la Demande du Gouverncment Français

**Published:** 1834-04-01

**Authors:** 


### XIII.

### Traité de la Vaccine, redigé sur la Demande du Gouvernement Francais. Par *M. Bousquet.*

Passing over the introductory matter of this volume, we shall endeavour to glean some of the most interesting and instructive particulars on a subject, which at present engages so much of the attention, not only of medical men, but also of the governments of mighty nations. It was in the year 1815 that the first distinct case of small-pox, occurring in a person who had been properly vaccinated, was made known in France by the Central Committee; and the fears which then began to be entertained respecting the inefficacy of vaccination, as a safeguard against the small-pox, were unfortunately much increased in 1818, when a very formidable epidemic of this latter disease broke out in most parts of Europe, and attacked a very considerable number of those who had hitherto been deemed almost quite secure. The epidemic of Paris in 1825, and especially that of Marseilles in 1828, were, however, the most unfavourable to the credit of the Jennerian discovery in the public estimation, and many persons foolishly permitted themselves to become as sceptical of any good being ever derived from it, as some of its early partisans had been too sanguine in their praises upon its first introduction. The Minister of the Interior, M. Martignac, viewing the question as one of national importance, applied to the Academy of Medicine for information, to enable Government to determine, whether any new public measures should be taken for the eradication of that most grievous pestilence, the small-pox, and to fix what these measures should be. M. Bousquet, who had been for ten years secretary of the Vaccination Committee, was entrusted with the task of drawing up an ánswer to the Minister's interrogatories. Fortunately for medical science, he has not limited his researches merely to the solution of the precise question submitted to him, but has carefully and extensively examined many others; such as the subject of the varioloid or varioliform eruptions, which, by modifying the features of the genuine small-pox, have had the effect of misleading not a few medical writers. As to the description of the genuine original cow-pox "petite verole, ou picote des vaches," given us by Jenner, M. Bousquet regards it as very imperfect and unsatisfactory. He also dissents from Jenner's opinion, that the grease (eaux aux jambes) in horses is the exciting cause of the pock in cows; it having been supposed that the virus of the one was communicated to the other animal, by the country people who had charge of both. The facts which have been adduced to prove this are, in his opinion, by no means conclusive.

In spite of the assertions of some recent authors, M. Bousquet informs us that the genuine cow-pox, in the cow, is of exceedingly rare occurrence, and that, therefore, the few opportunities of studying its genuine properties and relations, have hitherto much retarded our knowledge of this very interesting subject. It appears from our author's statements, that the success in producing artificially the cow-pox, by vaccination, varies a good deal at different times; but, whether this unsteadiness be connected with particular seasons and states of the atmosphere, has not yet been satisfactorily determined. In the month of May, 1830, by far the greater number of the vaccinations performed by our author entirely failed; and, at the very same time, M. Nanche was equally unsuccessful in his practice. Again, in the following year, the results were almost as unsatisfactory; but the data furnished this time were more precise, and they were more accurately noted. Thus, it was observed that, in the vaccinations performed from the 30th of April to the 7th May, the course of the vesicles advanced so quickly to their complete maturity, that any one might have supposed that they were of ten or twelve, instead of only eight days' standing. There was nothing unusual noticed in the vesicles on the child which had furnished the virus, and the children vaccinated were in all respects healthy. Now five children were vaccinated with the contents of the above-mentioned premature vesicles, and, on the eighth day afterwards (14th), the vesicles in four of them were found to be imperfect and ill-formed; while, in the fifth, there were four vesicles on each arm, and these vesicles as much advanced as on the children from whom the matter had been taken. The very reverse of all this was observed on the 17th of the month—the vesicles were then so small and tardy, that they seemed not to have passed the fifth day of vaccination.

In 1832, M. Gonville made similar observations; his vaccinations were quite successful in the months of April and May, but less so in June, and at length they were so unfortunate in July and August, that almost every operation required to be repeated two or three times. The cause of these variations in the action of the cow-pox virus resides, M. Bousquet thinks, in those mysterious, but, in all probability, potent influences, which Sydenham and Baillon have so forcibly illustrated, and proved to exist in the atmosphere; as to our own opinion, we have no doubt that cow-pox, like almost every other disease, especially such as are of a febrile character, are subjected to, and modified by, the operation of what has been denominated, of late years, by the term "medical constitutions." That the hygrometric, thermometric, and other states of the air, play an important part in the formation of morbific agents, will be admitted by almost every one, although the established data on this most curious topic are hitherto scanty and imperfect. Several instances might be adduced to prove, that extremes of heat and of cold have often impeded the right development of the vaccine vesicle; and we know that, in some very warm climates, the medical men find much difficulty in preserving a supply of the virus.

There is a piece of advice given by M. Bousquet in performing the operation, which it may be useful to mention:—The lancet, or whatever instrument is used for introducing the virus, should be held with its point sloping down, so that the virus may fall off by its own weight into the wound; and the skin ought to be kept on the stretch while the puncture is made, for thus the lips of the cut, returning on themselves, retain the virus better. The mere form of the vesicle is of much less consequence, in determining whether it be genuine or spurious, than its development and progress; in the former, it is more slow and gradual, seldom making any appearance until the end of the third, or the beginning of the fourth day, whereas, in the latter, it is much more rapid, and has probably arrived at its maturity, while, in the other, it is still very imperfectly formed. We are not, however, to neglect altogether the outward characters of the vesicle; for the central depression, indeed, may be regarded as the anatomical type of the true, proper cow-pox vesicle—it is much less distinct, and may be even quite awanting, in the false or spurious one: besides this character, in the latter, it consists usually of one cyst only, and not of the cellular or honeycomb structure of the former—hence, the one is drained off by a single puncture, whereas the other requires several, to discharge its contents completely. The progress of the genuine vesicle is almost invariably and uniformly the same, although the period of incubation sometimes exceeds considerably the period of three or four days. Instances have been known, wherein the first set of punctures (supposing that the operation has been repeated) did not develope themselves until the second set was made; and again, in other instances, some of the punctures produced their specific irritation much more slowly than others. Occasionally, although rarely, the eruption is not confined to the seat of the punctures, and a few scattered vesicles make their appearance on different parts of the surface. In the course of M. Bousquet's inquiries, he satisfied himself that the contents of the vesicle, if drawn as soon as it makes its appearance, are quite as active and as fit for the purposes of inoculation, as when taken at any future period of its progress. We may, therefore, without the least hesitation, assert that the virus can never be "too young or unripe;" and that it is only an ignorant prejudice of many authors, when they recommend us to await the more complete formation of the pock, in order that we may be more assured of the efficacy of its contents. The latter part of M. Bousquet's work is occupied with the consideration of the more general questions on the subject of the cow-pox, more especially in relation to its effects on the human body, in connexion with those of the small-pox. He regards the cow-pox as a strictly eruptive fever, which, instead of being opposed to and inconsistent with that of variola, as the vague and illogical descriptions of many authors might lead us to suppose, is of a nature analogous to it; indeed, it is the result of this very analogy, that the two diseases have so striking a reciprocity of influence on each other. It is most satisfactory to learn, that the conviction which the very extended researches have left upon his mind, of the paramount importance of vaccination, as an object of national, as well as of individual concern, remains unchanged, or rather has been greatly confirmed. The chief source of the apprehensions which have prevailed, of late years, exists, according to his opinion, in medical men having not rightly understood the analogy which holds between the cow-pox and the small-pox, on the one hand, and between the latter and the varioloid and varicellar eruptions, on the other. With justice he puts the question—"Why should cow-pox be supposed or expected to possess exclusively the prerogative of never suffering a relapse, when we know that the congeneral diseases of variola, varioloides, and varicella, are subject to this accident?" Had we reasoned aright, we should have anticipated, *à priori*, to have met with occasional cases of small-pox supervening to vaccination, on the mere ground, that we were aware that a second attack of genuine variola itself happens now and then, although the general rule will not be gainsayed, that this disease affects the system but once.

It is from having allowed themselves to entertain too high an opinion of vaccination at first, that so many persons now undervalue it far below its deserts. Some observers, who admit the full efficacy of the Jennerian discovery, have attempted to account for its more frequent failure within the last 10 years, by supposing that the virus has degenerated, and that it will be necessary to derive it directly from the primary source, viz. the vesicles on the cow. But this opinion is quite conjectural, and we have a very easy method of testing its correctness, by comparing the vesicles which we observe after vaccination, in the present day, with the descriptions and drawings of those left us by the original authors. M. Bousquet maintains, that such a comparison will afford results not at all favourable to the notion of the degeneration of the vaccine virus, in consequence of its repeated transmission through human bodies. It has been asserted that the cicatrices left by the vesicles, of late years, have been less distinct and indented than they used to be; but whether this be true or not, it may be reasonably doubted whether the depth of the scar stands in any appreciable relation to the preservative constitutional effects of the virus; every day we meet with cases, in which a person having very faint scars, or not even a trace of them, resists the variolous poison, while others, who have them well marked, are subjected to its influence. On the whole, there is no good reason for the opinion, that the vaccinal virus has lost any of its properties; and, moreover, it is quite unnecessary, says our author, to renew it directly from the cow—a fortunate circumstance, because, up to the present moment, all attempts to do so have proved unsuccessful (?)

